# Enhancement of Fracture Toughness of NiTi Alloy by Controlling Grain Size Gradient

**DOI:** 10.3390/nano15020125

**Published:** 2025-01-16

**Authors:** Kai Huang, Zhongzheng Deng, Hao Yin

**Affiliations:** 1 School of Civil Engineering, Wuhan University, Wuhan 430072, China; 00030505@whu.edu.cn; 2 Department of Mechanical and Aerospace Engineering, The Hong Kong University of Science and Technology, Hong Kong, China

**Keywords:** NiTi alloys, fracture toughness, fracture behavior, grain size, gradient

## Abstract

Fracture toughness is a critical indicator for the application of NiTi alloys in medical fields. We propose to enhance the fracture toughness of NiTi alloys by controlling the spatial grain size (GS) gradient. Utilizing rolling processes and heat treatment technology, three categories of NiTi alloys with distinct spatial GS distributions were fabricated and subsequently examined through multi-field synchronous fracture tests. It is found that the one with a locally ultra-high GS gradient (20 nm−3.4 μm) has significantly enhanced fracture toughness, which is as high as 412% of that of the normally distributed nano-grains with an average GS of 8 nm and 178% of that of the coarse-grains with an average GS of 100 nm. Theoretical analysis reveals that in such a gradient structure, phase transition in the coarse-grained matrix greatly absorbs the surface energy of subcritical and stable propagation. Meanwhile, the locally non-uniform GS distribution leads to deviation and tortuosity of the crack path, increasing the critical fracture stress. Furthermore, the nanocrystalline clusters distributed in the form of network nodes reduce the stress intensity factor due to their higher elastic modulus compared to the coarse-grained matrix. This work provides guidance for developing new gradient nanostructured NiTi alloys with high fracture toughness.

## 1. Introduction

NiTi alloys have been widely used in medical fields (such as cardiovascular stents, endodontic files, and braided catheters) owing to their outstanding superelasticity and biocompatibility [1,2,3]. However, the presence of inherent defects within these NiTi devices can lead to localized stress concentrations, potentially causing device failure and unforeseen harm to the human body; the investigation of fracture performance of NiTi alloys has received extensive attention [4,5,6,7,8,9]. Researchers have discovered substantial variations in fracture toughness and failure behavior among commercially available polycrystalline NiTi alloys, leading to concerns regarding their stability and reliability in practical applications [10,11,12,13]. Understanding the underlying causes of these variations is crucial for developing more robust NiTi alloys, and will greatly save the operational costs of related medical instruments [14,15]. Grain size (GS) and grain distribution, as the most important characteristics of polycrystalline NiTi alloys, are considered to be the main factors affecting mechanical properties, especially fracture toughness [16,17,18].

The commercial coarse-grained (CG) NiTi alloys with GS larger than 1 µm have large recoverable strains (>6%), significant latent heat, and considerable fracture toughness, but have relatively low tensile strengths [19,20,21]. The experimental results of fracture tests present that the majority of CG NiTi alloys are prone to ductile failure, while a few percent encounter mixed failure [22,23]. When the average GS is reduced to the nanometer level, the mechanical properties and phase transition (PT) characteristics of the NiTi alloy undergo notable changes, especially below 60 nm [24,25,26,27,28]. Hong et al. indicated that the significant increase in the density of the grain boundary of nanocrystalline NiTi leads to grain refinement strengthening, enhanced tensile strength and transformation stress, linearized stress–strain relation with shrinking hysteresis, broadened superelasticity window, and improved cyclic stability [29,30]. However, the transformation strain and fracture toughness are weakened, leading to diminished superelasticity and an increased probability of brittle failure [31,32,33]. Latest studies on nano-grains (NGs) with notches or cracks indicate that the fracture toughness of NGs does not exhibit superior potential compared to that of CGs, regardless of plane stress or plane strain states [34,35]. Aslan et al. observed a consistent decline in the maximum stress intensity factor (SIF) with decreasing GS under plane stress conditions [36]. Leitner et al. found that the fracture toughness of equal-channel-angular extruded NiTi did not improve despite a 1.5-fold and a 2.6-fold increase in transformation stress and martensitic yield stress, respectively, compared to that of the original CG with GS of 100 µm [37]. Xie et al. investigated the fracture behavior of NiTi alloys with GS ranging from 17 µm to 45 µm using molecular dynamics simulations. They found that as the grains become coarsened, the duration of subcritical crack growth and the critical stress intensity factor (*K*_IC_) increase, and become more tortuous [38]. These studies primarily concentrate on average GS, neglecting the effects of grain distribution. However, since static crack propagation in polycrystals often extends across several or even hundreds of grains, the impact of the GS gradient along the crack path within this range cannot be ignored. It is essential to reveal how the GS gradient influences fracture toughness and fracture behavior at various spatial scales.

Developing preparation processes to achieve precise control at the micro-/nanoscales is a key step for the investigation. At present, severe plastic deformation (SPD) remains a prevalent method for refining the GSs of NiTi alloys. Through SPD, methods such as forging, equal channel angular extrusion, high-pressure torsion, cold-rolling, drawing, and canning compression can yield amorphous or nanocrystalline structures [39,40,41]. Grain growth can be controlled through appropriate aging or annealing methods. Peterlechner et al. have found that SPD can produce retained nanocrystallites with high dislocation density, significant distortion, and amorphous phases [42]. Since the amorphous phase is more stable, a higher temperature is needed for early recovery and crystallization [43,44,45,46]. In subsequent annealing, nanoclusters with high activation energy can be formed after recrystallization. Meanwhile, retained nanocrystals recover and continue to grow at lower temperatures as dislocations are eliminated. The varying dislocation concentrations and distortions in nanocrystals result in different activation energies [26,47,48,49,50]. As a result, uneven grain growth occurs, creating local GS gradients through heat treatment. Based on this understanding, in order to regulate GS gradient and grain distribution, there are two essential points: 1. modifying the SPD process to control the extent of nanocrystalline distortion, dislocation density, and amorphous content; 2. adjusting the annealing temperature and the heating rate to control grain growth. For instance, in order to create a locally high GS gradient, symmetrical rolling can be replaced with asymmetrical rolling, which can generate higher internal shearing stress, resulting in a prestressed hybrid cold-rolled nanocrystalline structure [51,52]. This structure is composed of substructures with high dislocation density and distributed nanocrystalline nuclei, surrounded by amorphous and ultrafine nanocrystals. This leads to variations in activation energy for grain growth within local regions [22,44,45,53]. When such a structure undergoes annealing at variable heating speeds, the different growth rates lead to temporal and spatial gradients in the recovery and recrystallization processes, resulting in locally high GS gradients.

In this work, we proposed new methods for controlling grain growth in NiTi alloys and engineered GS gradients across various spatial scales. We have successfully prepared samples exhibiting normal GS distributions, unidirectional GS gradient, and locally high gradient GS. The fracture toughness and fracture behavior of these samples were investigated by in situ multi-field synchronous fracture tests. To capture the small-scale instantaneous nonlinear deformation at the crack tip, we have developed high-speed microscopic Digital Image Correlation (DIC) techniques. In addition, high-precision infrared thermal imaging technology was employed to record temperature variation under subcritical propagation and unstable failure. Through theoretical analysis, we have discussed the toughening mechanism of a locally high GS gradient. This study provides insights for developing new nanocrystalline NiTi shape memory alloys with enhanced reliability and fracture toughness.

## 2. Materials and Methods

### 2.1. Material Preparation

The material received was a NiTi plate with a thickness of 4.0 mm. The composition was determined using a JEOL JSM-IT700HR equipment equipped with an UltimMax 65 Energy Dispersive Spectroscopy (EDS) across 15 distinct regions, yielding an average composition of Ti-50.36at% Ni. Preparation processes were developed to achieve the following three categories:

(1) Normal GS Distributions

**Figure 1:**
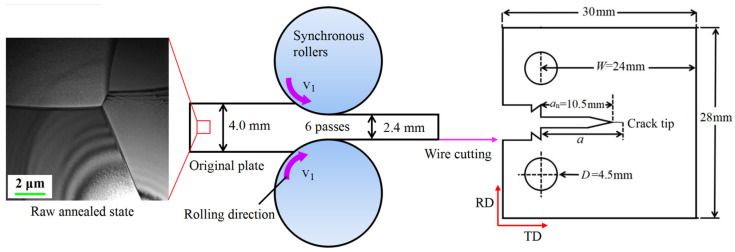
Using synchronous cold-rolling, the received CG plate is repeatedly rolled to reduce its thickness to 2.4 mm, resulting in grain refinement. Subsequently, CT specimens can be obtained from the cold-rolled plates through wire cutting, with the specific dimensional information provided.

The received plate was subjected to homogenization annealing at 800 °C for 1 h, followed by quenching in water. Subsequently, 6 passes of synchronous cold-rolling reduce the thickness to 2.4 mm (Figure 1). To prevent excessive local GS gradients, a pre-aging method at 220 °C for 4 h was used to minimize differences in activation energies, followed by a second annealing to control grain growth. Compact tension (CT) samples were cut from the plates in accordance with the ASTM E1820 standard (the same as the other two categories). The key dimensions are provided on the right side of Figure 1.

(2) Macroscopic Unidirectional GS Gradient

Firstly, the received plates were cold-rolled using the same method illustrated in Figure 1. Subsequently, the cold-rolled CT samples were annealed at 220 °C for 2 h to eliminate organizational defects and homogenize the activation energies. Then, one end of the CT sample was heated using a heat-conducting clamp, while the other end was cooled using circulating water. Once the temperature stabilized, a temperature gradient of 80–550 °C was formed from the cooling boundary to the heating boundary, as measured by thermocouples.

(3) Locally High GS Gradient

**Figure 2:**
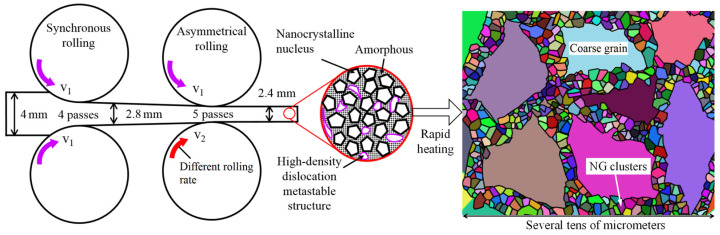
Material process for achieving a locally high GS gradient. By employing synchronous rolling to reduce thickness and refine grains, followed by enhancing shear strain through asymmetrical rolling, a complex mixed structure is achieved that contains nanocrystalline nuclei, high-density nano-metastable structures, and amorphous phases. Locally high-gradient samples are produced through rapid annealing, a process characterized by non-uniform growth during recrystallization, with different colors indicating distinct grains.

The received plate was cold-rolled to a thickness of 2.8 mm using synchronous rolls over 4 passes. Afterward, 5 passes of asymmetrical rolling further reduce the thickness to 2.4 mm. Two rolled samples were rapidly heated from 100 °C to 600 °C at rates of 5 °C/min and 30 °C/min, respectively, and subsequently cooled in air. These samples, which exhibited high GS gradients in localized areas, are designated as HG1 and HG2, respectively. The preparation process is depicted in Figure 2.

Transmission Electron Microscopy (TEM) observations were conducted using a JEOL JEM-F200 instrument (operating at 200 kV) to examine the microstructures of various samples. Microstructural analyses at the micron scale were employed using a JEOL JSM-7900F Scanning Electron Microscope (SEM) operating at 20 kV with a step size of 0.2 μm. Electron backscatter diffraction (EBSD) was utilized to analyze the distribution of grain orientations. Circular samples weighing 20 mg, with a diameter of 3.0 mm and a thickness of 0.4 mm, were cut from the CT samples and tested using a Mettler Toledo Differential Scanning Calorimeter (DSC). The samples were subjected to DSC experiments at a heating/cooling rate of 10 °C/min within the range of −80 °C to 150 °C. XRD experiments were conducted by Bruker D8 ADVANCE with a Cu-Ka radiation source in the range of 5° < 2 h < 90°. To determine the mechanical properties, micro dumbbell-shaped samples cut from the CT samples were stretched at a rate of 1 mm/min using an Instron 5969 testing machine.

### 2.2. Testing Methods

**Figure 3:**
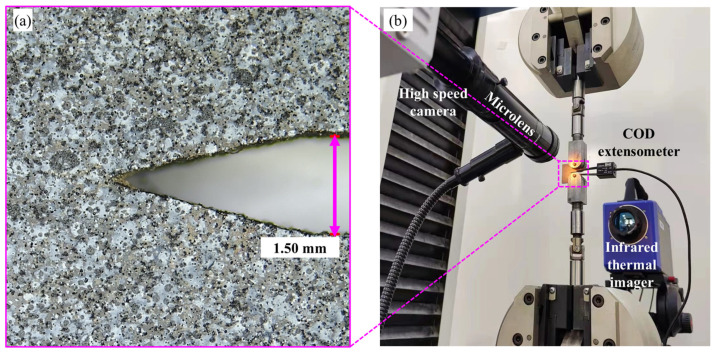
(**a**) A high-quality DIC surface featuring fine speckle patterns of varying sizes and gray levels from the tested CT sample. (**b**) Multi-field synchronous fracture testing system, including details on sample installation and instrument information.

The surfaces of the CT samples were polished to remove the mechanically damaged layer. Their notches were refined using tungsten steel blades with an 800-mesh diamond until the root radii were reduced to less than 50 μm. The crack lengths of two opposite surfaces were measured by a tool microscope to ensure that the difference was less than 0.02 mm. An MTS Landmark 370 material testing machine was used to prefabricate cracks. In total, 5 Hz sinusoidal cyclic loading was implemented with a load ratio of *R* = *P*_min_/*P*_max_ = 0.1. The prefabricated crack length was controlled within the 0.45 < *a*/*W* < 0.55 range. One surface, intended for infrared detection, was evenly coated with matte black paint. For the DIC detection surface, an approximately 10 μm thick layer of white paint was applied. Subsequently, an airbrush was employed to apply black speckles to the surface, resulting in finely distributed speckles with varying shades of gray, as shown in Figure 3a. A multi-field synchronous monitoring system was developed to enable the full-field measurement of deformation and temperature variation, as illustrated in Figure 3b. The system comprises a Photron SA-5 high-speed camera equipped with a 125× microscopic lens, a universal testing machine featuring a COD gauge, and a FLIR SC7700M high-frequency infrared camera. The CT specimens were stretched at 2 mm/min until they fractured. The Correlated Solutions Vic-2D software (https://www.correlatedsolutions.com/vic-2d, accessed on 3 December 2024) was used to analyze the full-field strain. The original crack length, *a*_0_, can be measured using a microscope in accordance with ASTM E1820.

### 2.3. Evaluation Method for Fracture Toughness

The crack tip stress field can be expressed as follows [54]:

(1) $$\left\{\begin{array}{c} \sigma_{x}=\frac{K_{I}}{\sqrt{2\pi r}}\cos\frac{\theta }{2}(1-\sin\frac{\theta }{2}\sin\frac{3\theta }{2})\\ \sigma_{y}=\frac{K_{I}}{\sqrt{2\pi r}}\cos\frac{\theta }{2}(1+\sin\frac{\theta }{2}\sin\frac{3\theta }{2})\\ \tau_{xy}=\frac{K_{I}}{\sqrt{2\pi r}}\sin\frac{\theta }{2}\cos\frac{\theta }{2}\cos\frac{3\theta }{2} \end{array}\right.$$

*K*_I_ is determined as follows (see ASTM E1820):

(2) $${K_{I}=\frac{P}{{BW}^{0.5}}f}_{2}(\frac{a}{W})$$

(3) $$f_{2}\left(\frac{a}{W}\right)=\frac{\left(2+\frac{a}{W}\right)[0.886+4.64\frac{a}{W}-13.32{\left(\frac{a}{W}\right)}^{2}+14.72{\left(\frac{a}{W}\right)}^{3}-5.6{\left(\frac{a}{W}\right)}^{4}]}{{\left(1-\frac{a}{W}\right)}^{\frac{3}{2}}}$$

where *B* is the thickness, *a* is the crack length, and *W* is the distance from the pin hole to the right edge (see Figure 1). Passing through the origin of the P-COD curve and using 95% of the elastic slope as the intersection line, the point where it intersects the P-COD curve is *K*_Q_. When the condition meets the criterion of plane strain, the *K*_Q_ is *K*_IC_.

For CGs, energy dissipation occurs through PT, martensitic reorientation, and plastic deformation. Instability propagation can only occur when the rate of strain energy release exceeds the energy dissipation. In such cases, considering the *J* integral,

(4) $$J=J_{e}+J_{p}={\left[\frac{P}{{BW}^{0.5}}\times f_{2}\left(\frac{a}{w}\right)\right]}^{2}\left[\frac{\left(1-\vartheta^{2}\right)}{E}\right]+\left[\frac{\eta_{p}U_{p}}{B\left(W-a_{0}\right)}\right]\{1-[\frac{(0.75\eta_{p}-1)\Delta a}{W-a}]\}$$

(5) $$\eta_{p}=2+0.522(1-a_{0}/W)$$

where $J_{e}$ is the elastic component and $J_{p}$ is the plastic component. The specific drawing method of the *J*-Δ*a* curve and determination of the critical value $J_{IC}$ can be referred to Haghgouyan et al. [55]. $K_{J_{Ic}}$($=\sqrt{EJ_{IC}}$) is used to calculate the extrapolated equivalent value of *K*_IC_.

## 3. Results

### 3.1. Results of GS Distributions

#### 3.1.1. Normal GS Distributions

**Figure 4:**
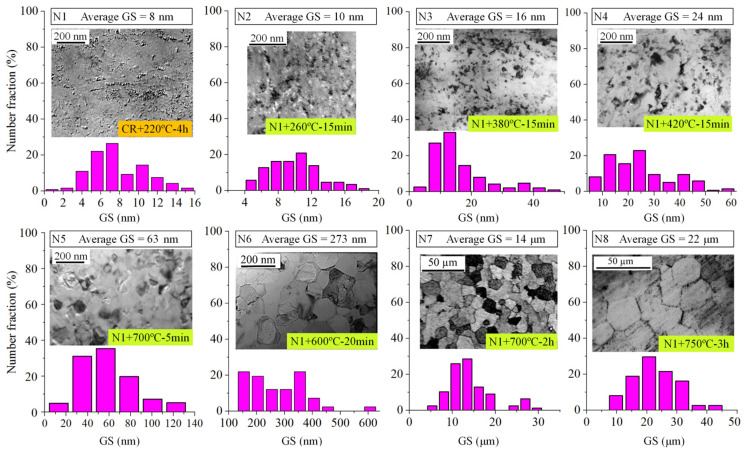
Microstructure characterization results of various samples with normal GS distributions: bright field images, statistical results of grain size distribution, and the corresponding annealing methods.

According to the material preparation for normal GS distributions described in Section 2.1, a series of samples with normally distributed GSs were prepared through different annealing treatments, which were labeled as N1 to N8. The corresponding heat treatments, TEM results, and GS statistics for these normally distributed samples are shown in Figure 4, presenting a series of normal distributions of different GS ranges. It is observed that as the temperature rises and the annealing time extends, the average GS consistently increases. Sample N1 predominantly consists of fragmented ultrafine grains, with a primary GS distribution of 6–10 nm. Low-temperature aging decreases defect density, homogenizes activation energy, minimizes local features, and has a minimal impact on grain growth. Annealing at temperatures ranging from 380 °C to 420 °C consists of two stages: recovery and recrystallization. In areas of high local deformation, subgrains form and then merge and grow incompletely, as illustrated in samples N3 and N4. At annealing temperatures exceeding 600 °C, crystalline nuclei rapidly form and grow toward the surrounding distorted regions through interface movement, resulting in the formation of undistorted equiaxed grains. The gradual and uniform growth of grains occurs almost simultaneously through grain boundary migration and mutual engulfment. It is evident that as the annealing temperature increases, the GS distribution approaches a normal state, resulting in reduced size variations, as demonstrated in samples N5, N7, and N8.

The DSC results presented in Figure S1a,b indicate that the PT capacities of the CG samples are significantly higher than those of the NG samples. Samples HG1 and HG2 exhibit a mixed phase of B_2_ (austenite) and B_19′_ (martensite), and their PTs are highly suppressed over a broad temperature range [56,57,58]. Samples N3 and N6 undergo a two-stage PT from the B_2_ phase to the R phase and then to the B_19′_ phase. Samples N7 and N8 are stable austenite phases at room temperature, and their PT modes are one-step processes in both heating and cooling. Figure S1c reveals that as average GS increases, the intensity of the B_2_ main peak consistently rises, while the martensite peak gradually diminishes. Concurrently, both the Ni_4_Ti_3_ and Ni_3_Ti phases begin to emerge.

#### 3.1.2. Macroscopic Unidirectional GS Gradient

**Figure 5:**
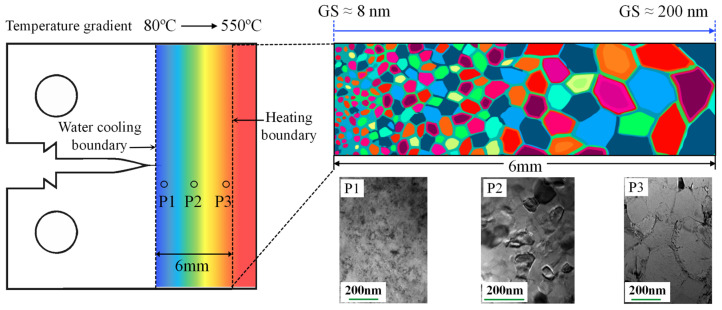
Schematic illustration of the temperature gradient across the CT sample and the distribution of the selected region. The TEM results at the selected positions of the sample GL reveal the macroscopic unidirectional GS gradient.

The CT sample depicted in Figure 5, with GS increasing from 8 nm at the crack tip to 200 nm at the distal end, is labeled as GL. The temperature gradient and GS distribution, along with the TEM results at the selected positions (P1, P2, and P3) of sample GL, are also illustrated in Figure 5, where the unidirectional GS distribution of 8−200 nm was formed within a 6 mm span. On the contrary, the CT sample where the GS decreases from 200 nm at the crack tip to 8 nm at the distal end is labeled as GR.

#### 3.1.3. Locally High GS Gradient

The statistics of PT temperatures for typical samples are presented in Table S1. The microstructure of sample HG1 is displayed in Figure S2. It is observed that there are mainly CG with an average GS of 12 μm encircled by smaller grains (Figure S3a). The grain boundaries (GBs) with misorientation angles below 5° account for approximately 30%. The orientation distribution function (ODF) map (Figure S4a) predominantly shows a strong <001>//RD, with secondary strong <101>// normal direction (ND) and <101>//TD (transverse direction) textures. These features indicate that the orientation is biased towards a <001> Gaussian texture.

**Figure 6:**
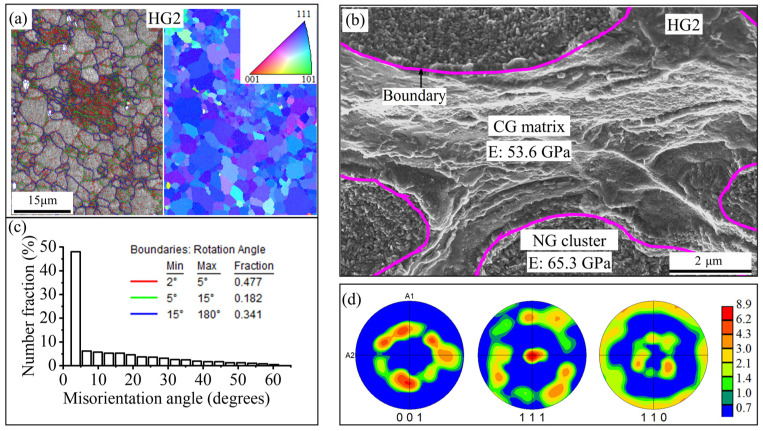
SEM observation and analysis of the sample HG2: (**a**) the SEM morphology with a corresponding IPF map shows fine grains dispersing within CGs, forming a dense network structure; (**b**) at higher magnification, NGs are reticulated around CGs in the form of clusters, resulting in an ultra-high GS gradient; (**c**) statistics on GB misorientation angles indicate the small-angle GBs account for the vast majority; (**d**) pole figures (PFs) indicate that the texture of the sample HG2 is close to the {111} Brass type.

**Figure 7:**
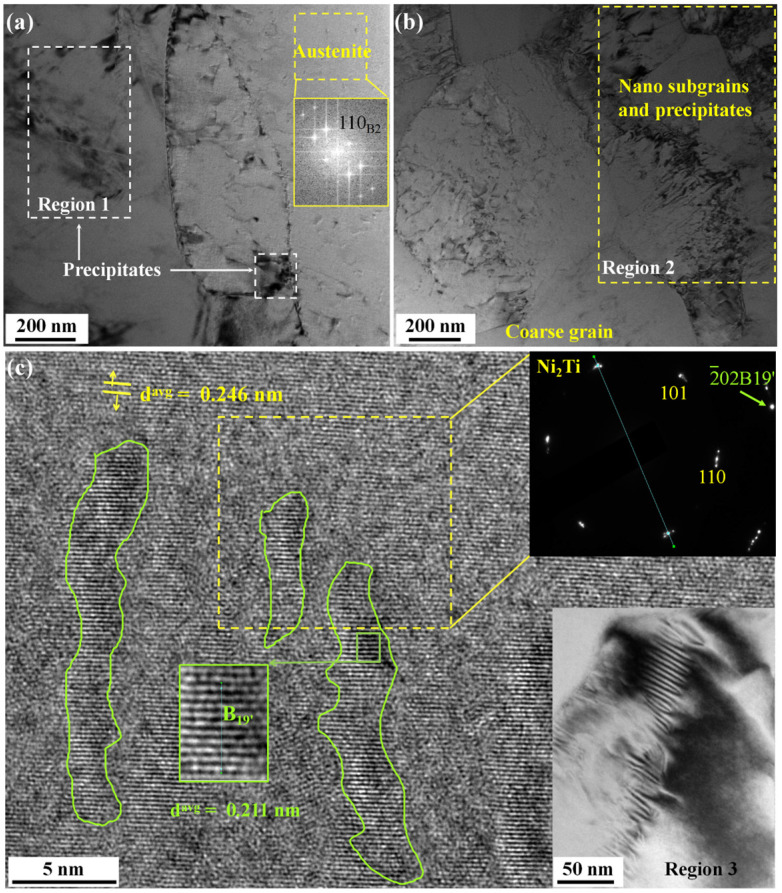
Microstructural characterization of three representative regions selected from the sample HG2. (**a**) Several coarse grains are interspersed with many nano subgrains and precipitated phases located at the grain boundaries and within the interior of the grains. Region 1 contains a small amount of Ni_2_Ti precipitates in the annealed B_2_ austenite matrix. (**b**) Dense nano subgrains and precipitated phases form clusters around larger crystals. (**c**) Ni_2_Ti precipitates (yellow dashed frame) coexist with austenite nanocrystalline subgrains, mixed with a small amount of elongated B_19′_ phase (green solid lines).

The microstructure of sample HG2 is shown in Figure 6a,b. It is observed that NGs (20 nm) are reticulated around CGs (average GS of 3.4 μm) rather than being severed. Misorientation angles below 5° account for up to 50% as seen in Figure 6c. Many nanocrystals and subgrain boundaries form dense networks on the CG substrate. The corresponding inverse pole figure (IPF) map shows the orientation is biased towards <111>. In conjunction with Figure S4b and Figure 6d, it is indicated that the texture is a mixture type of {111} <uvw> Brass, Residual rolled (S), and Copper. Figure S5a confirms that both HG1 and HG2 are basically austenite at room temperature. Sample HG1 shows a two-step PT mode (B_2_−R−B_19′_). There are small amounts of Ni_4_Ti_3_ and Ni_3_Ti precipitates in both samples HG1 and HG2 from Figure S5b. The TEM characterization results in Figure 7 further reveal a variety of precipitate sizes and the formation of B_2_-B_19′_-Ni_2_Ti mixed clusters at multiple sites. In Region 1, B_2_ austenite CG matrices are interspersed with many Ni_2_Ti precipitates located at the grain boundaries and within the grain interiors. The precipitates and nano subgrains form clusters in some areas. The precipitated phase Ni_2_Ti is compatible with the distorted martensite, and they interlock with each other to form large subgrains.

### 3.2. Fracture Testing Results

**Figure 8:**
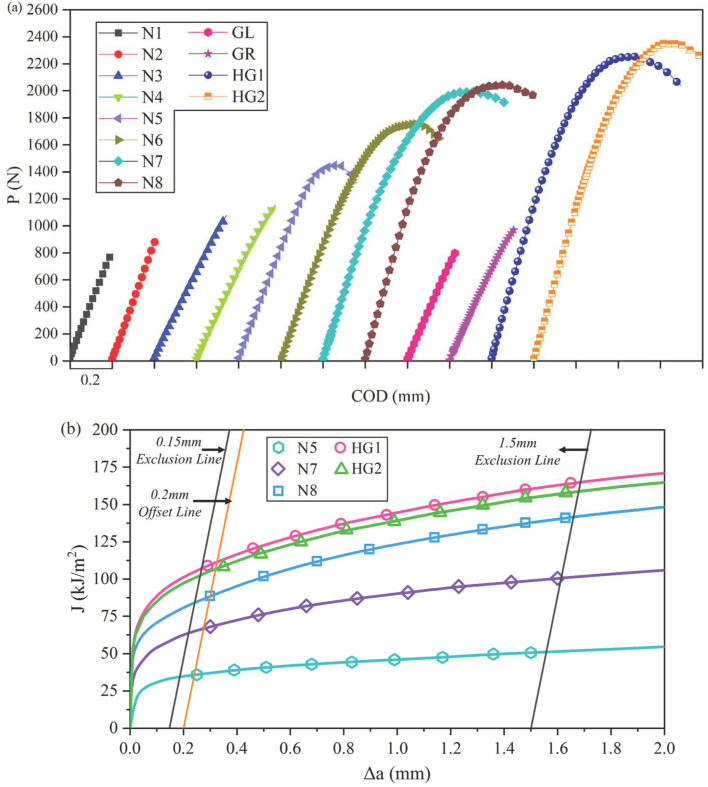
(**a**) *P*-*COD* curves of different categories of samples. (**b**) Comparison of the *J*-Δ*a* curves of typical resilient samples.

Figure 8a shows the *P*-*COD* curves for the three categories of samples. It is found that within 8–24 nm (N1–N4), PT is highly inhibited and the crack energy absorption rate is lower than the strain energy release rate. The curves approach linear elastic states, with no significant yielding observed near the maximum load, thus meeting the criteria for determining plane strain fracture toughness. Consequently, within this range, *K*_IC_ (=*K*_Q_) increases monotonically with GS. When GS rises more than 60 nm (N5–N8), two stages emerge on the *P*-*COD* curve: subcritical expansion (from the elastic limit to the maximum load) and unstable expansion. The measurement of fracture toughness should, therefore, employ the ductile *J*_IC_ method. For samples GL and GR, their fracture forces are only slightly higher than that of sample N1. From the characteristics of the curves, the subcritical propagation distance of the microcrack is notably less than the gradient GS distribution distance in samples GL and GR. The energy dissipation zone is limited to the grains near the crack tip. Therefore, the design of a millimeter-scaled GS gradient does not achieve a satisfactory enhancement of fracture force. The samples HG1 and HG2 show significantly higher fracture forces and more stable subcritical propagation compared to the other types of samples. According to the *J*-Δ*a* curves (Figure 8b) and the specific results shown in Table 1, it is evident that the locally high GS gradients possess larger equivalent *K*_IC_ than the other types. The equivalent *K*_IC_ of sample HG2 is 84.5 $\mathrm{MPa}\sqrt{m}$, which is as high as 412% of that for the normal distributed nanocrystallites with an average GS of 8 nm and 178% of that for 100 nm. Therefore, the locally high GS gradient indeed improves fracture toughness.

### 3.3. Thermal Coupling Fracture Behaviors

**Figure 9:**
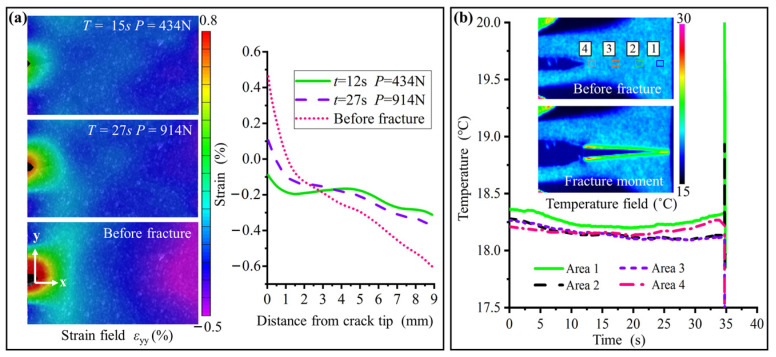
Thermomechanical fracture behavior of sample N2: (**a**) The DIC images and the strain (*ε*_yy_) distribution under various load conditions. (**b**) The thermal images at the fracture moments and the average temperatures of the four selected areas over time. The instantaneous fracture, minimal strain, and temperature variation at the crack tip indicate the typical brittle characteristics.

Figure 9a shows the DIC images and strain (*ε*_yy_) distribution near the crack tip of sample N2 under various loading conditions. Figure 9b presents the thermal images captured at the moment of fracture, along with the average temperatures of the selected areas over time. It is shown that there is no significant subcritical propagation prior to fracture. As COD increases, the strain at the crack tip increases, while the strain at the far end decreases. The maximum strains at both ends remain below 0.6%. Upon reaching the maximum COD, an instantaneous instability failure occurs, causing the crack to propagate completely through the material, which demonstrates a clear characteristic of brittleness. Due to the high yield stress and highly suppressed PT, the size of the plastic zone is extremely small, resulting in relatively minor temperature variations.

**Figure 10:**
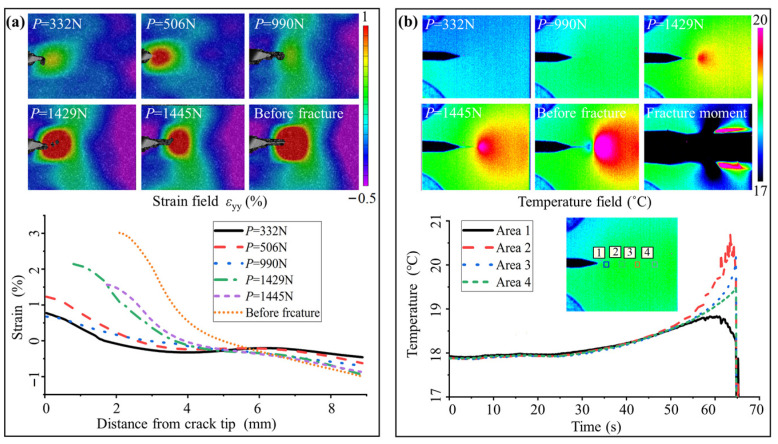
Fracture behavior of sample N5: (**a**) the DIC images and the corresponding strain distributions; (**b**) the thermal images and the average temperatures of the four selected areas over time. Strain oscillations occur during subcritical expansion, while temperature accumulation arises from PT and plastic deformation during unstable expansion.

Figure 10 shows the thermomechanical fracture behavior of sample N5. In the initial loading stage, crack propagation occurs slightly in subcritical propagation. PT consumes surface energy, resulting in decreases in strain at new locations at each new crack. As COD increases, the strain oscillates with subcritical propagation, accompanied by temperature oscillations. During the unstable expansion, heat continues to accumulate at the crack tip, while a tiny cooling area appears at the wake. The distance of unstable propagation is about 1.8 mm. Before fracture, the maximum temperature change is 2.7 °C, and the peak strain of the crack tip is 3.0%. These behaviors indicate that the increase in average GS leads to an increase in energy consumption by uncompleted PT.

**Figure 11:**
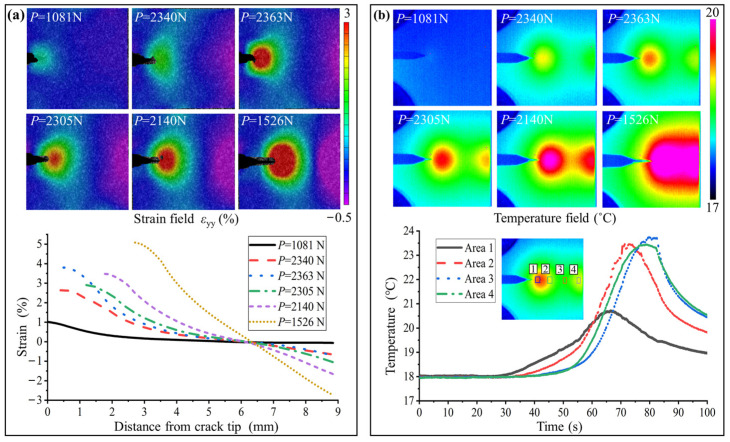
Fracture behavior of sample HG2 at different loading moments. (**a**) The DIC images and the corresponding strain distributions. (**b**) The thermal images and the average temperatures of the four selected areas with time. The crack continues to propagate steadily with large temperature accumulations.

Figure 11 shows the thermomechanical fracture behavior of sample HG2. It is seen that the size of the PT zone at the crack tip is significantly larger than that of the other samples. During unstable propagation, the end far from the crack tip experiences a continuous rise in temperature as COD grows because of compressive stress-induced PT. Therefore, there are two PT-induced temperature-rising zones simultaneously with crack propagation, which gradually merge with thermal conduction until the hot zone expands to one entire block zone. The distance of the subcritical propagation is more than 4.0 mm. The crack continues to propagate steadily without any penetrating damage. At 1526 N, the average temperature of the hot zone is about 6 °C higher than that of the substrate, and the peak strain of the crack tip reaches 5.2%. The results indicate that among the three categories, the sample with ultra-high GS distribution not only has the highest fracture toughness but also exhibits the most stable crack propagation performance.

### 3.4. Fracture Characteristics

**Figure 12:**
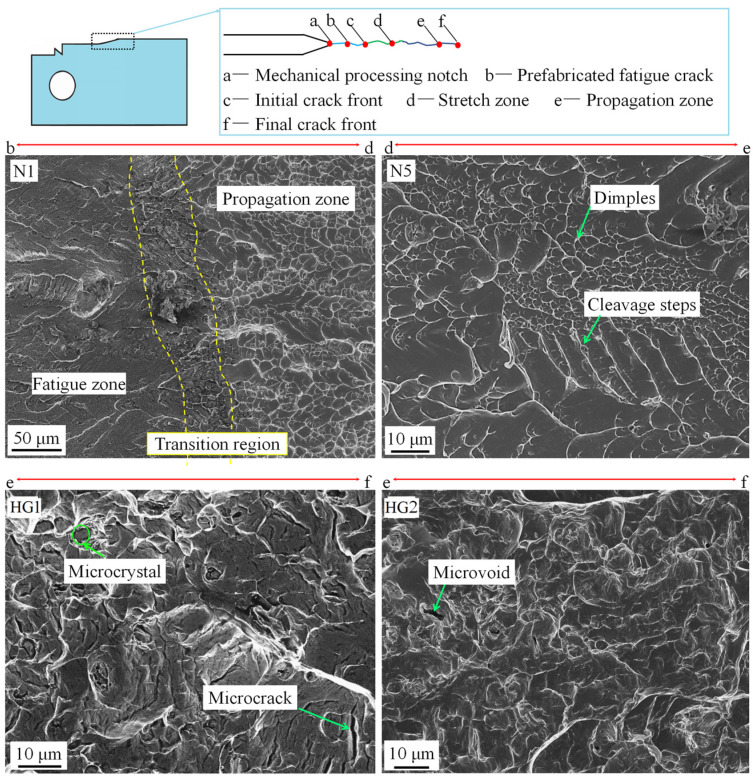
Schematic diagram of different stages of cracks and the SEM fracture morphologies at selected stages on the surfaces of typical samples.

A schematic diagram illustrating the different stages of crack development and SEM fracture morphologies at selected positions on the surfaces of typical samples is presented in Figure 12. It is observed that the fatigue zone of sample N1 displays a typical river pattern with cleavage steps, belonging to brittle transgranular fracture [59]. The propagation zone reveals certain pure shear features on a microscale and the fracture characteristics of micro-pore aggregation on a macroscale. There is a transition zone with an average width of 30 µm, which contains many pores, defects, and fishbone patterns with larger dimples. This detail reveals that many defects accumulated at the crack propagation end, caused by high internal stress at the beginning of the static tensile zone, which considerably lowers the fracture stress below its intrinsic critical value. For sample N5, the coexistence of cleavage steps and dimples displays mixed fracture features. Adjacent grains are collectively bound around anti-sliding grain boundaries, causing the internal interface between grains to suffer plastic deformation. Local shear planes are concentrated on adjacent grain planes, forming cluster grains embedded in a sliding environment. The plastic slip interfaces composed of multiple grains form shear planes, resulting in a dimple size much larger than the GS. For sample HG1, there were some micro-pores and cracks that appeared, which were caused by damage to the low-index crystal planes of large-sized grains. There are also some dimples with sizes of 2–15 µm randomly arranged at the macro level. Based on the EBSD results in Figure S2, it can be concluded that the fracture toughness of the sample is limited due to the Gaussian texture. For sample HG2, there are few micro-voids and a large number of micrometer-sized dimples in the propagation zone, showing characteristics of ductile intergranular fracture. Compared to HG1, HG2 has smaller-sized microcracks with lower density, a larger filamentary area, and a smaller radiation area. Therefore, the ductile features of sample HG2 are more pronounced compared to the other samples.

## 4. Discussions

**Figure 13:**
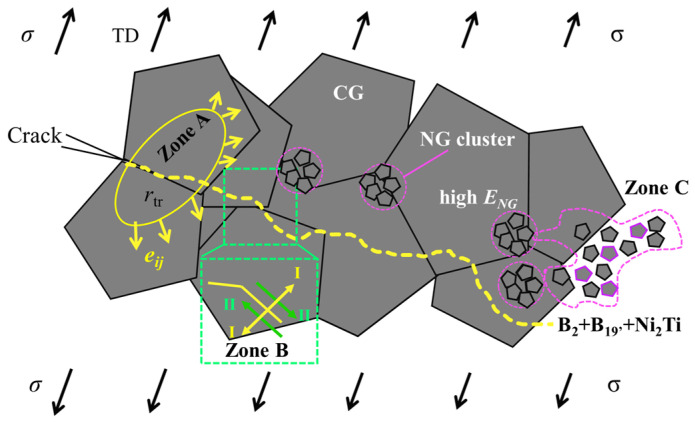
Schematic diagram of the subcritical propagation path of sample HG2. There are three types of zones affecting the crack path. Zone A: The CG region consumes the surface energy by PT. Zone B: Deviation and tortuosity of the crack path caused by locally non-uniform GS distribution. Zone C: NG clusters with mixed precipitates change the modulus of the front tip. The characteristics of these three regions collectively influence the trajectory and energy of subcritical crack propagation.

The toughening mechanism of the locally high GS gradient is elucidated in Figure 13. There are three main mechanisms: (1) stress-induced PT in the CG region consumes the surface energy, (2) deviation and tortuosity of the crack path caused by high GS gradient and locally non-uniform GS distribution lead to an increase in the critical fracture stress, (3) NG clusters with high elastic modulus are distributed in the form of network nodes in the CG matrix, reducing the SIF.

### 4.1. Toughening Enhancement by Stress-Induced PT in CG Region

**Figure 14:**
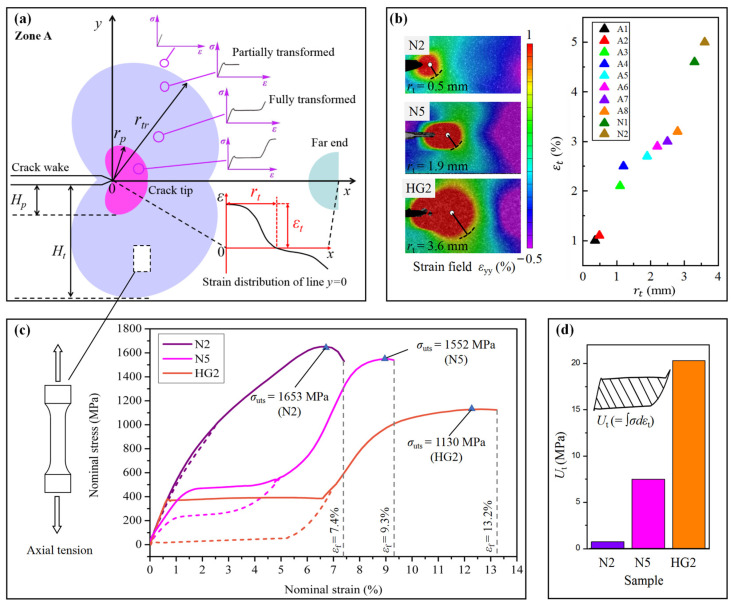
(**a**) Schematic diagram of the stress field of the CGs in region A of Figure 12. The PT and plastic deformation near the crack tip absorb a significant amount of crack opening energy. A high strain gradient is formed from the crack tip to the opposite end. Determination method of the radius ($r_{t}$) and strain magnitude ($\varepsilon_{t}$) of the PT toughening zone. (**b**) Comparison of strain fields before fracture for three typical samples, along with a comparison of the $r_{t}$ and $\varepsilon_{t}$ values for all the samples. (**c**) The ultimate tensile strength, fracture strain, and hysteresis can be determined by the stress–strain response of micro-bulks at the crack tip under uniaxial tension. (**d**) Comparison of hysteresis ($U_{t})$ for typical samples.

The toughening extent caused by PT of the crack tip mainly depends on dimensionless parameters $\alpha =\sigma^{tr}\varepsilon^{tr}/{\left[(\sigma^{tr}\right)}^{2}E^{A}]$ (ratio of dissipated to stored energy) where $\sigma^{tr}$ is the transformation stress, $\varepsilon^{tr}$ is the transformation strain, and $E^{A}$ is the Young’s modulus of austenite [60]. A higher *α* value is beneficial for enhancing toughness. Therefore, the lower $\sigma^{tr}$ and higher $\varepsilon^{tr}$ of CG mainly cause toughness enhancement. The toughness enhancement ΔK for steady-state crack propagation can be described by the following Equation [61]:

(6) $$\Delta K=K_{app}-K_{Ic}=\sqrt{{K_{Ic}}^{2}+\frac{2E^{A}}{\left(1-\vartheta^{2}\right)}[\int_{0}^{H_{t}}U_{t}\left(y\right)dy]}-K_{Ic}$$

where $H_{t}$ is the height of the PT zone at the crack tip, and $U_{t}(=\int \sigma d\varepsilon_{t})$ is the specific mechanical energy dissipation by PT. Figure 14a shows the stress field of the CG state of region A in Figure 13. The $r_{t}$ is the radius of the PT toughening zone in the *y* = 0 direction, which is positively correlated with $H_{t}$. As shown in Figure 14b, both $r_{t}$ and $\varepsilon_{t}$ of sample HG2 are significantly larger than those of the other samples. Figure 14c shows the ultimate tensile strength, fracture strain, and hysteresis of typical samples, which were determined by the stress–strain response of micro-bulks at the crack tip under uniaxial tension. It is evident that sample HG2 exhibits the highest transformation stress and fracture strain, as well as the lowest yield stress. The dashed line represents the stress–strain response of fully transformed PT. Figure 14d illustrates the large hysteresis area of sample HG2 compared to the other samples. According to Equation (6), the high hysteresis and the radius of sample HG2 indicate that the PT in the CG regions of the network greatly absorbs the surface energy generated by crack opening.

### 4.2. Toughening Enhancement by Locally Non-Uniform GS Distribution

Cracks normally propagate along the paths with low-index crystal planes and small orientation differences in the CG region [49]. However, as hindered by local nanocrystallites with high PT stress and yield stress, the crack prefers to propagate along the CG interface with lower PT stress. This motion leads to a deviation or tortuosity configuration of the crack path, where the crack mode changes from Type I to mixed type (Zone B in Figure 13). According to the strain energy release rate criterion (G-criterion), which assumes that composite cracks interact on an elastic component so that the elastic *J*-integral and elastic strain energy release rate become equal, the *J*-integral is used as a critical property to determine the onset of stable crack growth. Thus, Equation (7) for the elastic mixed-mode interaction is applicable for stable crack motion.

(7) $$J_{i}=G_{i}=G_{I}+G_{II}+G_{III}$$

(8) $$J_{i}=G_{i}=G_{I}+G_{II}+G_{III}$$

For pure mode I, the plane strain fracture toughness expression is

(9) $$G_{IC}=G_{I}=(1-\vartheta^{2})\frac{K_{IC}^{2}}{E}$$

Substituting Equation (9) into (8) gives the mixed-mode fracture criterion

(10) $$K_{IC}^{2}=K_{I}^{2}+K_{II}^{2}+\frac{K_{III}^{2}}{1-\vartheta }$$

**Figure 15:**
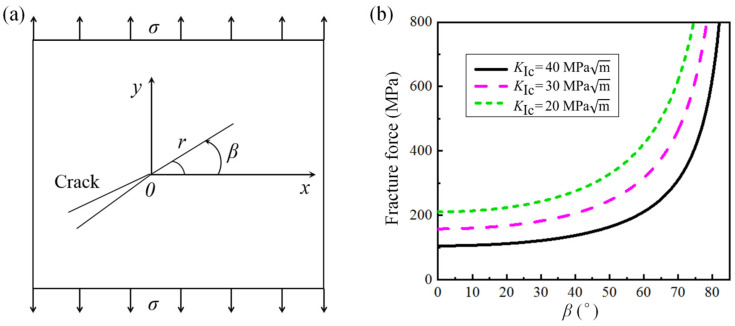
(**a**) The mixed-typed cracks with inclination angle *β*; (**b**) the effect of crack inclination angle *β* on critical fracture stress at different fracture toughness levels.

Considering the mixed-mode I/II configuration shown in Figure 15a, where the stress tensor components are defined by

(11) $$\left.\begin{array}{c} \sigma_{x}={\sigma sin}^{2}\beta \\ \sigma_{y}={\sigma cos}^{2}\beta \\ \tau_{xy}=\sigma \sin\beta \cos\beta \end{array}\right\}$$

The applied *SIF*s are

(12) $$K_{I}=\sigma_{y}\sqrt{\pi a}={\sigma cos}^{2}\beta \sqrt{\pi a}$$

(13) $$K_{I}=\sigma_{y}\sqrt{\pi a}={\sigma cos}^{2}\beta \sqrt{\pi a}$$

With the G-criterion as an elliptical model, Equation (10) becomes

(14) $${\left(\frac{K_{I}}{K_{IC}}\right)}^{2}+{\left(\frac{K_{II}}{K_{IIC}}\right)}^{2}=1\;(ellipse)$$

(15) $$K_{I}^{2}+{C_{e}K}_{II}^{2}=K_{IC}^{2}\;(ellipse)$$

where $C_{e}$ is a constant, substituting Equations (12) and (13) into Equations (14) and (15) so that the fracture stress is a function of fracture toughness and inclined crack angle:

(16) $$\sigma_{f}=\frac{K_{IC}}{cos\beta \sqrt{\pi a}}\sqrt{\frac{1}{{cos}^{2}\beta +C_{e}{sin}^{2}\beta }}$$

According to Equation (16), Figure 15b is obtained to show the relationship between the fracture stress and inclination angle *β* under various fracture toughness levels. It is seen that the fracture stress threshold increases with *β*. Therefore, the deviation and tortuosity of the crack path caused by locally non-uniform GS distribution lead to an increase in fracture stress.

### 4.3. Toughening Enhancement by NG Clusters

**Figure 16:**
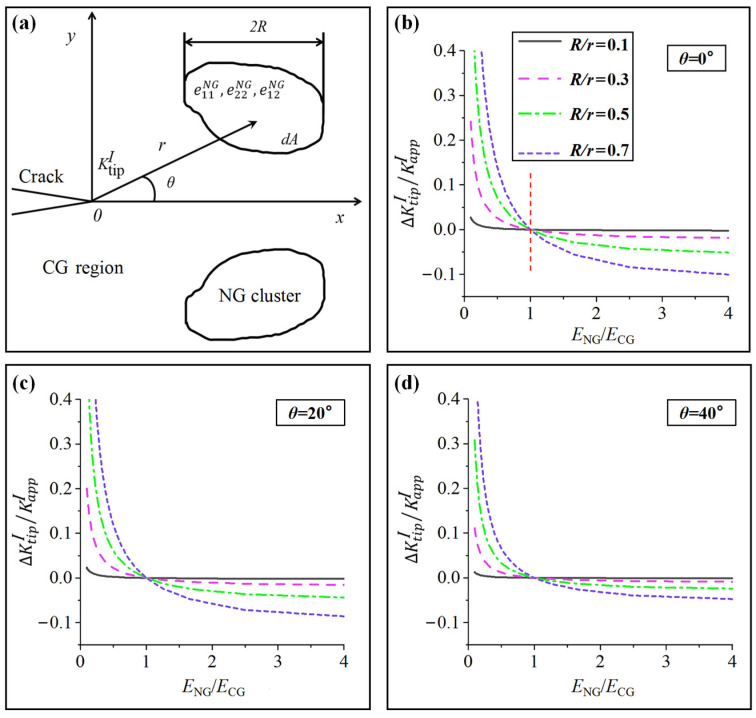
(**a**) Distribution of the micro-elements represents the NG clusters in front of the CG region of the crack tip. The effect of the ratio between the radius of the NG cluster and its distance from the crack tip on ${\Delta K}_{tip}^{I}/K_{app}^{I}$ under various *θ* angles: (**b**) *θ* = 0°; (**c**) *θ* = 20°; (**d**) *θ* = 40°.

NG clusters are distributed in the CG network nodes (Zone C in Figure 13). The cluster’s size is relatively small compared to the crack length, and its transformation stress is much higher than that of the CG matrix. An NG cluster can be approximately considered as a circular inclusion with a radius of *R*, as shown in Figure 16a. The effective elastic modulus of the CG and NG regions are *E*_CG_ and *E*_NG_, respectively. The difference in Poisson’s ratio is small and can be simplified as the same. The change in *SIF* is ${\Delta K}_{\mathrm{tip}}^{I}\;$($=K_{\mathrm{tip}}^{I}-K_{app}^{I}$). When ${\Delta K}_{tip}^{I}$ is less than 0, it indicates that the inclusion has toughened the matrix, while when ${\Delta K}_{tip}^{I}$ is greater than 0, it reduces the toughness. The change in *SIF* caused by the NG cluster is as follows:

(17) $${dK}_{tip}^{I}=\frac{1}{2\sqrt{2\pi }}\frac{E_{CG}}{1-\vartheta^{2}}r^{-\frac{3}{2}}\Omega_{1}(e_{\alpha \gamma }^{NG},\theta )dA$$

(18) $$\Omega_{1}\left(e_{\alpha \gamma }^{NG},\theta \right)=cos\frac{3\theta }{2}\left(e_{11}^{NG}+e_{22}^{NG}\right)+\frac{3}{2}sin\theta sin\frac{5\theta }{2}\left(e_{22}^{NG}-e_{11}^{NG}\right)+3sin\theta cos\frac{5\theta }{2}e_{12}^{NG}$$

where $e_{11}^{NG},e_{22}^{NG}$, and $e_{12}^{NG}$ are the strains at the crack tip (r,θ) without stress and non-zero strain at the tip of mode I crack:

(19) $$\left.\begin{array}{c} e_{11}=\frac{\left(1+\upsilon \right)K_{app}^{I}}{E\sqrt{2\pi r}}\cos\frac{\theta }{2}[\left(1-2\upsilon \right)-\sin\frac{\theta }{2}\sin\frac{3\theta }{2}]\\ e_{22}=\frac{\left(1+\upsilon \right)K_{app}^{I}}{E\sqrt{2\pi r}}\cos\frac{\theta }{2}[\left(1-2\upsilon \right)+\sin\frac{\theta }{2}\sin\frac{3\theta }{2}]\\ e_{12}=\frac{\left(1+\upsilon \right)K_{app}^{I}}{E\sqrt{2\pi r}}\sin\frac{\theta }{2}\cos\frac{\theta }{2}\cos\frac{3\theta }{2} \end{array}\right\}$$

Substituting $E_{CG}$ and $E_{NG}$ into Equation (19) and making a first-order approximation, the unconstrained elastic unmatched strain in the NG region can be expressed as follows:

(20) $$\left.\begin{array}{c} e_{11}^{CG}=\left(1+\upsilon \right)(\frac{1}{E_{NG}}-\frac{1}{E_{CG}})\frac{K_{app}^{I}}{\sqrt{2\pi r}}\cos\frac{\theta }{2}[\left(1-2\upsilon \right)-\sin\frac{\theta }{2}\sin\frac{3\theta }{2}]\\ e_{22}^{CG}=\left(1+\upsilon \right)(\frac{1}{E_{NG}}-\frac{1}{E_{CG}})\frac{K_{app}^{I}}{\sqrt{2\pi r}}\cos\frac{\theta }{2}[\left(1-2\upsilon \right)+\sin\frac{\theta }{2}\sin\frac{3\theta }{2}]\\ e_{12}^{CG}=\left(1+\upsilon \right)(\frac{1}{E_{NG}}-\frac{1}{E_{CG}})\frac{K_{app}^{I}}{\sqrt{2\pi r}}\sin\frac{\theta }{2}\cos\frac{\theta }{2}\cos\frac{3\theta }{2} \end{array}\right\}$$

Essentially, Equation (20) represents the PT strain without constraints in Equation (18). The increment of *SIF* caused by the unconstrained non-matched strain $e_{\alpha \gamma }^{CG}$ can be calculated by Equation (17):

(21) $${dK}_{tip}^{I}=\frac{1}{2\sqrt{2\pi }}\frac{E_{CG}}{1-\vartheta^{2}}r^{-\frac{3}{2}}\Omega_{1}(e_{\alpha \gamma }^{CG},\theta )dA$$

(22) $$\Omega_{1}\left(e_{\alpha \gamma }^{CG},\theta \right)=\left(1+\upsilon \right)(\frac{1}{E_{NG}}-\frac{1}{E_{CG}})\frac{K_{app}^{I}}{\sqrt{2\pi r}}[\left(1-2\upsilon \right)cos\frac{\theta }{2}cos\frac{3\theta }{2}+\frac{3}{4}{sin}^{2}\theta cos\theta ]$$

If the size of the NG cluster is given

(23) $${\Delta K}_{tip}^{I}=CK_{app}^{I}\int_{A}r^{-2}(cos\frac{\theta }{2}cos\frac{3\theta }{2}+\frac{3}{4\left(1-2\upsilon \right)}\frac{3}{4}{sin}^{2}\theta cos\theta )dA$$

where *C* is the coefficient related to the elastic modulus:

(24) $$C=\frac{1-2\upsilon }{2\pi \left(1-\upsilon \right)}(\frac{E_{CG}}{E_{NG}}-1)$$

When the angle between the inclusion and the crack tip extension line is relatively small, the contribution of the second term can be ignored, and ${\Delta K}_{tip}^{I}$ can be expressed as follows:

(25) $${\Delta K}_{tip}^{I}\approx CK_{app}^{I}\int_{A}r^{-2}cos\frac{\theta }{2}cos\frac{3\theta }{2}dA$$

*R*/*r* (0.1–0.7) and *θ* (0–40°) are substituted into Equation (25), adopting ${\Delta K}_{tip}^{I}/K_{app}^{I}$ to nondimensionalize *SIF* and elastic modulus ratio ($E_{NG}/E_{CG}$) as the variable. The result of the influence of the NG region on the CG matrix is shown in Figure 16b–d. As $E_{NG}/E_{CG}$ is greater than 1, for all the selected values of *R*/*r* at four angles, ${\Delta K}_{tip}^{I}$ are all less than 0, and the curve gradient monotonically decreases as the angle increases, indicating that the impact of $E_{NG}/E_{CG}$ on toughness is mainly in the small angle range. The toughening effect is proportional to the size of the NG cluster, as seen by the curve gradient’s monotonic increase with *R*/*r*. Due to the higher elastic modulus of NG clusters [56,62], ${\Delta K}_{tip}^{I}/K_{app}^{I}$ becomes lower. Therefore, the NG clusters distributed at small angles in the front of the crack tip do play a toughening role.

## 5. Conclusions

To investigate the impact of grain size (GS) distribution on the fracture performance of NiTi alloys and to enhance their fracture toughness, we developed three categories of NiTi alloys by controlling the GS gradient at different spatial scales: normal GS distributions, macroscopic unidirectional GS gradient at the millimeter scale, and locally high GS gradient network distributions at the micrometer scale. In the third category, the nano-grains or fine-grains are reticulated around coarse-grains in the form of clusters. The experimental results indicate that the sample with a locally ultra-high GS gradient (20 nm–3.4 μm) exhibits significantly enhanced fracture toughness, with an equivalent *K*_IC_ of 84.5 $\mathrm{MPa}\sqrt{m}$. This value is as high as 412% of that of the nanocrystallites with an average GS of 8 nm and 178% of that of the commercial coarse-grained NiTi with a GS of 100 nm. The toughening mechanisms associated with the locally high GS gradient are revealed through both experimental and theoretical analyses. The conclusions are as follows:

(1) The high degree of phase transition of coarse grains within the network greatly absorbs surface energy. In this case, both the radius of the phase transition zone, the strain of the core area, and the specific mechanical energy dissipation by PT are larger than any kind of normal distribution.
(2) The deviation and tortuosity of the crack path, caused by high GS gradient and locally non-uniform GS distribution, result in a change in the crack mode from Type I to a composite mode, which increases the actual fracture stress.
(3) The nanocrystalline clusters distributed at a small angle in front of the crack tip reduce the applied stress intensity factor due to their higher elastic modulus compared to the coarse-grained matrix in the network structure.

## Figures and Tables

**Table 1:** Comparison of mechanical properties and fracture toughness.

Category	Sample	Transformation Stress (MPa)	Yield Stress (MPa)	Transformation Strain (%)	Fracture Force (N)	*K*_IC_ ($\mathrm{MPa}\sqrt{m}$)	*K*_JIC_ ($\mathrm{MPa}\sqrt{m}$)
Normal	N1	N/A	N/A	N/A	785	20.5	N/A
	N2	N/A	N/A	N/A	908	23.7	N/A
	N3	559	2180	2.0	1178	30.7	N/A
	N4	535	1669	2.9	1156	28.8	N/A
	N5	460	1536	4.1	1446	N/A	47.3
	N6	433	1247	6.2	1753	N/A	59.9
	N7	292	1153	6.6	1994	N/A	67.2
	N8	287	1125	6.5	2044	N/A	75.3
Unidirectional gradient	GL	N/A	N/A	N/A	800	20.8	N/A
	GR	N/A	N/A	N/A	980	25.5	N/A
Locally high gradient	HG1	380	1178	6.4	2256	N/A	82.8
	HG2	377	1103	6.6	2363	N/A	84.5

## Data Availability

The data presented in this study are available upon request from the corresponding author.

## Supplementary Materials

The following supporting information can be downloaded at https://www.mdpi.com/article/10.3390/nano15020125/s1, Figure S1: Thermodynamic properties and phase characterization of the samples with normal GS distributions; Table S1. Statistics of phase transition temperatures of typical samples; Figure S2: Microstructure characterization of sample HG1; Figure S3: Grain size (GS) statistical results; Figure S4: The orientation distribution function (ODF) maps; Figure S5: Thermal performance and phase characterization of samples HG1 and HG2; Figure S6: TEM characterization results of two selected positions of region 1 in Figure 7. Selected position 1 represents the typical structure in the coarse-grained matrix.
