# Supplementary material for: Enhancement of Fracture Toughness of NiTi Alloy by Controlling Grain Size Gradient
Source: Nanomaterials (Basel). 2025 Jan 16;15(2):125. doi: 10.3390/nano15020125 (PMC11767600; doi:10.3390/nano15020125)
Supplement: Supplementary file 1 [file nanomaterials-15-00125-s001.zip › nanomaterials-3380573-supplementary.pdf]

Enhancement of fracture toughness of NiTi alloy by controlling grain size gradient

Kai Huang[1], Zhongzheng Deng[2,*] and Hao Yin[1,*]

[1] *Department of Engineering Mechanics, School of Civil Engineering, Wuhan University, Wuhan, China*

[2]*Department of Mechanical and Aerospace Engineering, The Hong Kong University of Science and Technology, Hong Kong, China*

Supplementary material

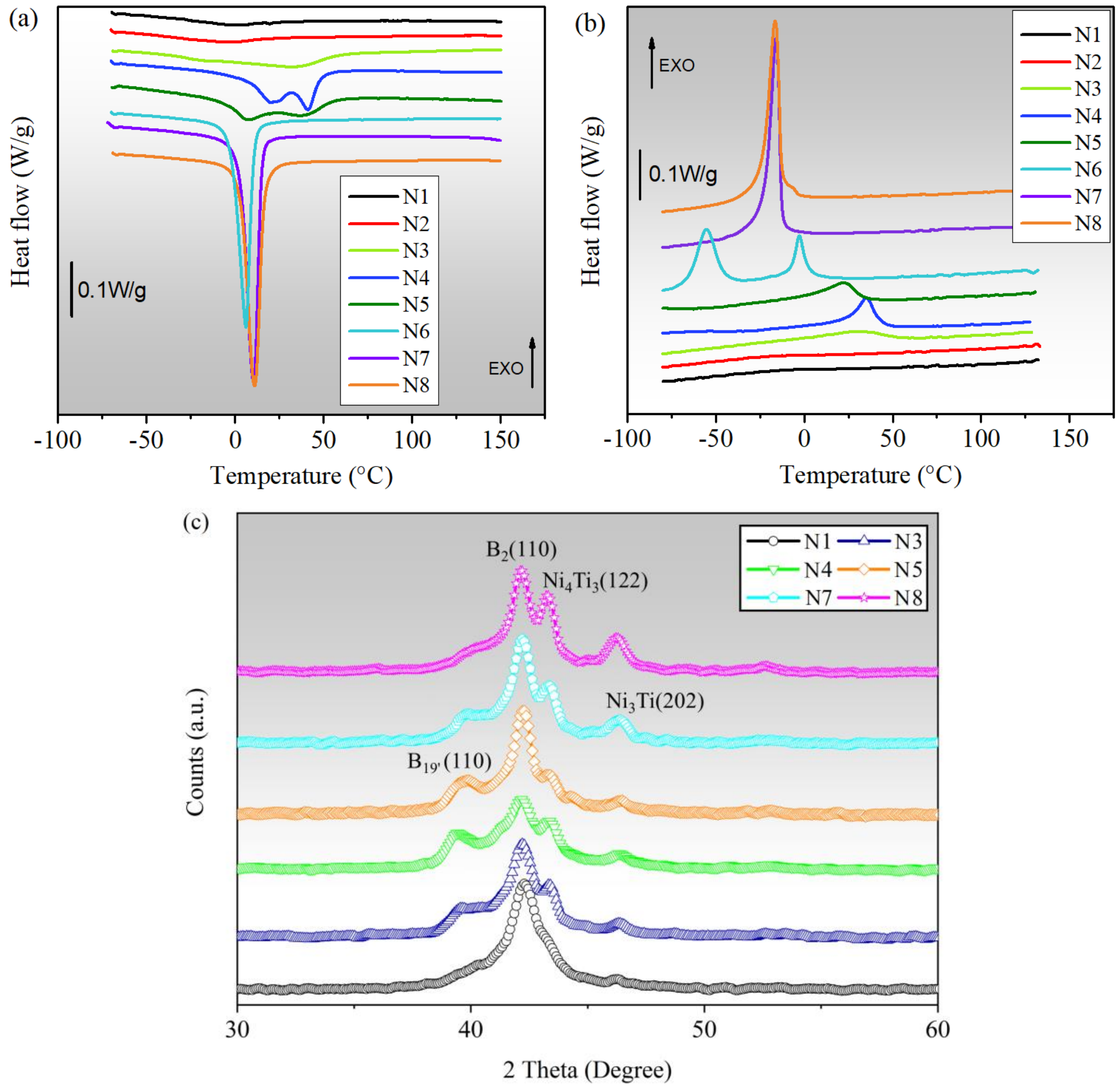

Figure S1. Thermodynamic properties and phase characterization of the samples with normal GS distributions: (a) Differential scanning calorimetry (DSC) curves of heating process; (b) DSC curves of cooling process; (c) X-ray diffraction results.

Table S1. Statistics of phase transition temperatures of typical samples

Category	Sample	A_s (ºC)	A_f (ºC)	M_s (ºC)	M_f (ºC)
Normal distribution	N1	—	—	—	—
	N2	—	—	—	—
	N3	—	56.8	51.4	—
	N4	7.9	48.4	44.0	22.0
	N5	-4.0	56.3	33.4	9.4
	N6	-4.5	12.6	3.5	-68.1
	N7	0.5	16.5	-11.0	-27.5
	N8	-1.4	18.0	-6.5	-27.0
Locally high gradient distribution	HG1	-6.1	10.3	-12.0	-56.1
	HG2	2.0	12.5	-15.5	-27.5

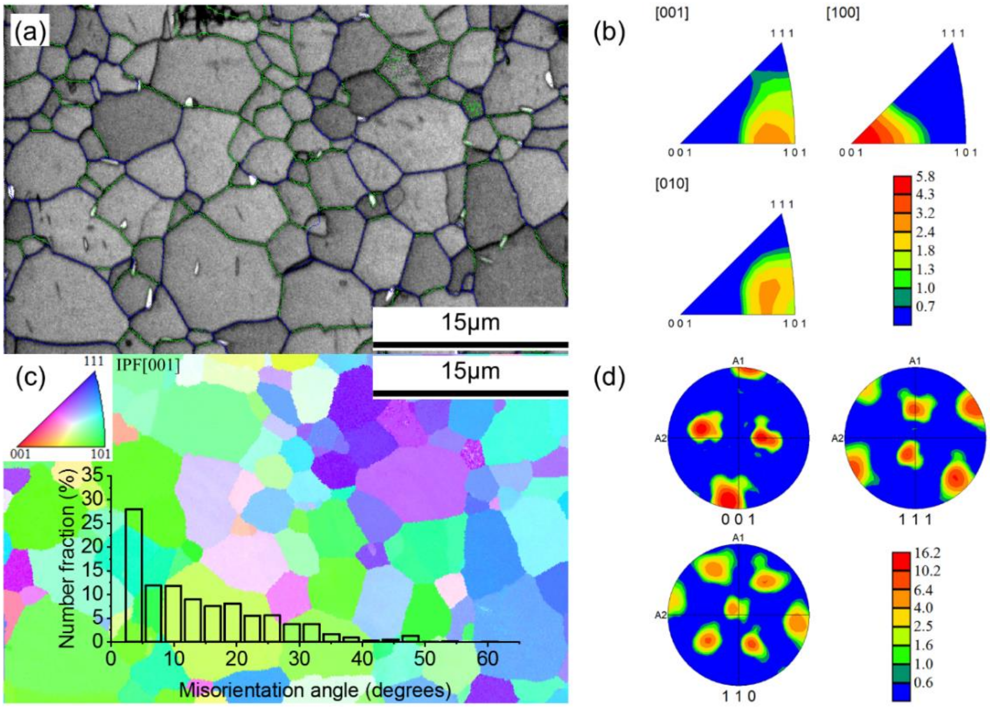

Figure S2. Microstructure characterization of sample HG1: (a) SEM morphology; (b) The corresponding inverse pole figure (IPF); (c) IPF maps and statistics of grain boundaries misorientation angles; (d) The corresponding pole figure (PF).

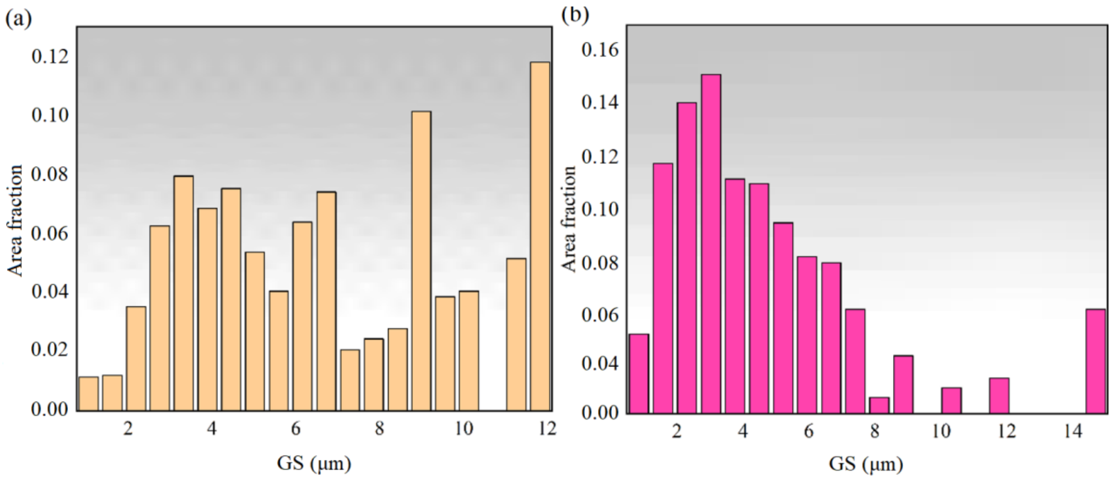

Figure S3. Grain size (GS) statistical results: (a) sample HG1 and (b) sample HG2.

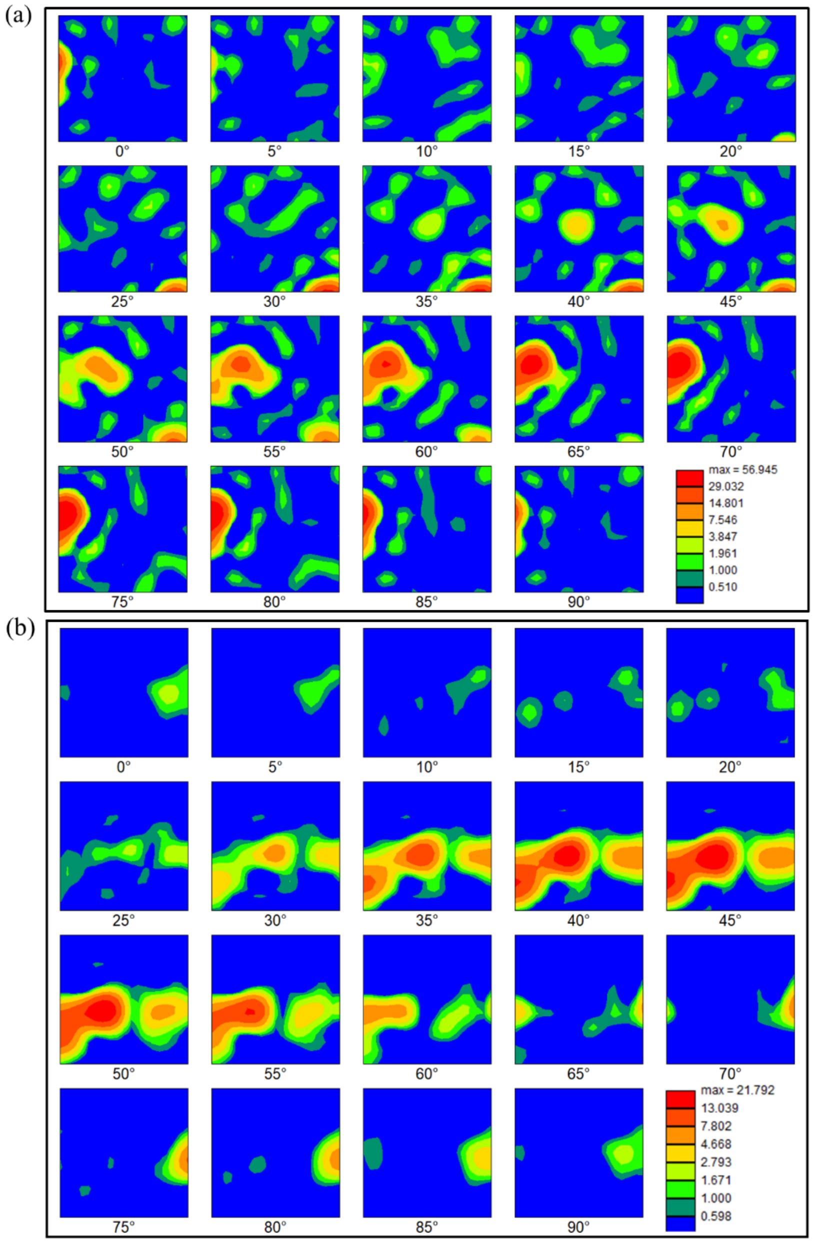

Figure S4. The orientation distribution function (ODF) maps: (a) sample HG1, (b) sample HG2.

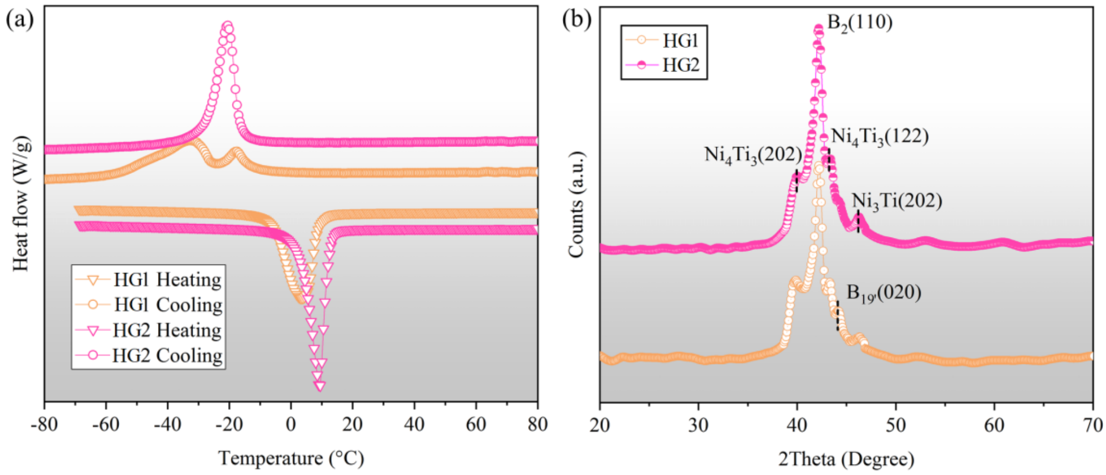

Figure S5. Thermal performance and phase characterization of samples HG1 and HG2: (a) DSC results. (b) XRD results.

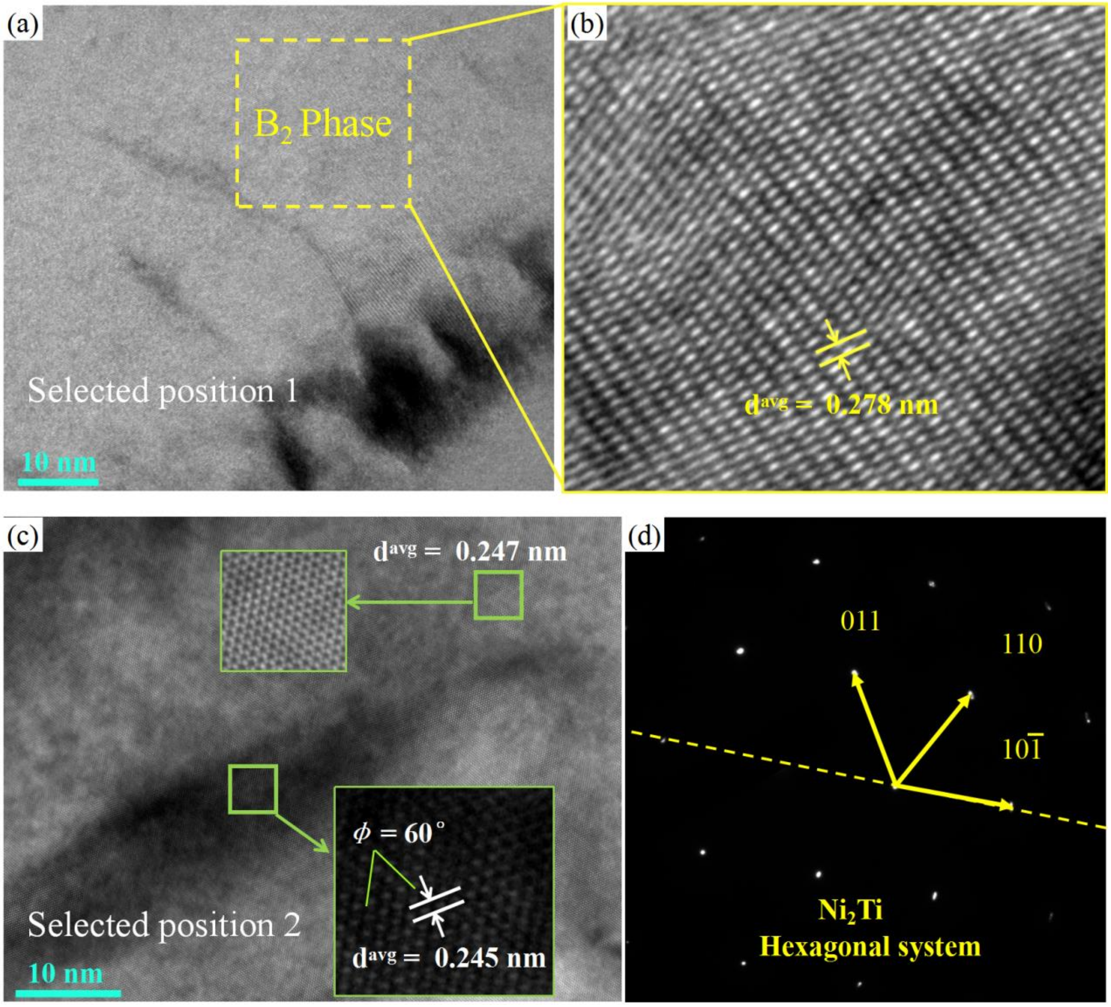

Figure S6. TEM characterization results of two selected positions of region 1 in **Figure 7**. Selected position 1 represents the typical structure in the coarse-grained matrix: (a) Austenite B_2 structure with interface of nano subgrains and boundaries; (b) The corresponding lattice stripes. Selected position 2 represents Ni_2Ti structures: (c) Bright field image and lattice stripes of the precipitation phase. The areas with darker contrast are the Ni_2Ti precipitates with larger distortion; (d) The corresponding selected area electron diffraction pattern show a typical hexagonal system of Ni_2Ti precipitate.
